# An integrative theoretical model to predict positive aspects of caregiving in dementia

**DOI:** 10.1093/geroni/igab046.2918

**Published:** 2021-12-17

**Authors:** Doris Yu, Sheung-Tak Cheng, Timothy Kwok

**Affiliations:** 1 University of Hong Kong, Hong Kong, Hong Kong; 2 The Education University of Hong Kong, The Education University of Hong Kong, Hong Kong; 3 The Chinese University of Hong Kong, Shatin, N.T., Hong Kong, Hong Kong

## Abstract

Family caregiving for dementia is the crucial informal care resource to buffer the associated disease burden. Whereas substantial research focused on ameliorating the caregiving burden through increasing their coping resources, least attention is placed on how to promotive their positive aspects of caregiving (PAC). This longitudinal exploratory study aimed at testingWhereas the perceived self-efficacy was further enriched in the context of good social sup an integrative theoretical model which attempts to explain the evolvement of PAC from the paradigm of stress and coping and existentialism. From to June 2017 to April 2020, we have recruited a total of 403 dementia caregivers from the a geriatric clinic in Hong Kong (mean age = 56.2, SD = 12.2; child-caregiver: 73.9%). About 61% of them were taking care of PwD of moderate to severe dementia. Validated instruments were used to measure the hypothesized model constructs. By using path analysis, it was found that PAC was evolved from two conditions, including i) perceived self-efficacy developed through active coping strategies for carers with good to moderate social support and ii) meaning-focused coping in the context of high religiosity, better social support and active coping. Data-model fit was evident by RMSEA = 0.023, CFI = 0.994, NFI = 0.968 and AIC = 97.762. The findings suggested that PAC was evolved from the interaction of the stress-coping and meaning-making process. Empowering carers for successful caregiving experience, facilitating them to make meaning in the process, enhancing good dyadic relationship and social support are crucial to cultivate PAC.

